# Risk factors for and outcomes of poststroke pneumonia in patients with acute ischemic stroke treated with mechanical thrombectomy

**DOI:** 10.3389/fneur.2023.1023475

**Published:** 2023-03-07

**Authors:** Ping Zhang, Lei Chen, Yi Jiang, Hui Yuan, Xuan Zhu, Minmin Zhang, Tao Wu, Benqiang Deng, Pengfei Yang, Yongwei Zhang, Jianmin Liu

**Affiliations:** Neurovascular Center, Changhai Hospital, Naval Medical University, Shanghai, China

**Keywords:** pneumonia, ischemic stroke, thrombectomy, outcome, inflammation

## Abstract

**Objective:**

The purpose of the study was to assess the risk factors for poststroke pneumonia (PSP) and its association with the outcomes in patients with acute ischemic stroke (AIS) due to large artery occlusion treated with mechanical thrombectomy (MT).

**Methods:**

Consecutive patients with AIS who underwent MT from January 2019 to December 2019 in the stroke center of Changhai Hospital were identified retrospectively. All of the patients were evaluated for the occurrence of PSP while in the hospital, and their modified Rankin scale (mRS) scores were assessed 90 days after having a stroke. Logistic regression analysis was conducted to determine the independent predictors of PSP, and the associations between PSP and clinical outcomes were analyzed.

**Results:**

A total of 248 patients were enrolled, of whom 33.47% (83) developed PSP. Logistic regression analysis revealed that body mass index (BMI) [unadjusted odds ratio (OR) 1.200, 95% confidence interval (CI) 1.038–1.387; *p* = 0.014], systemic immune-inflammation index (SII) (OR 1.001, 95% CI 1.000–1.002; *p* = 0.003), dysphagia (OR 9.498, 95% CI 3.217–28.041; *p* < 0.001), and intubation after MT (OR 4.262, 95% CI 1.166–15.581; *p* = 0.028) were independent risk factors for PSP. PSP was a strong predictor of clinical outcomes: it was associated with functional independence (mRS score ≤ 2) (OR 0.104, 95% CI 0.041–0.260; *p* < 0.001) and mortality at 90 days (OR 3.010, 95% CI 1.068–8.489; *p* = 0.037).

**Conclusion:**

More than one in three patients with AIS treated with MT developed PSP. Dysphagia, intubation, higher BMI, and SII were associated with PSP in these patients. Patients with AIS who develop PSP are more likely to experience negative outcomes. The prevention and identification of PSP are necessary to reduce mortality and improve clinical outcomes.

## Introduction

Mechanical thrombectomy (MT) has been proven to be effective for patients with acute ischemic stroke (AIS) due to large artery occlusion (1). Although most of these patients achieve complete recanalization after MT, many patients with AIS die of complications (2). The most common complication is pneumonia, which negatively affects clinical outcomes and increases the cost and duration of hospitalization (3). The prediction of poststroke pneumonia (PSP) remains challenging. No single biomarker pattern predicting PSP or outcome has been identified (3). The severity of stroke and dysphagia are considered risk factors for PSP (4). However, there is still no conclusive evidence regarding the risks and effects of PSP after MT (5). Whether thrombolysis before MT or anesthesia adds potential health risks for patients and increases PSP rates is still uncertain (6, 7). Therefore, it is necessary to investigate the risk factors for PSP and its association with outcomes in patients with AIS treated with MT. The goal of this study was to evaluate the predictive factors of PSP in patients with AIS with MT and the association between PSP and clinical outcomes.

## Methods

### Patient cohort

In this retrospective study, patients who presented at the Department of Stroke Center at Changhai Hospital between January 2019 and December 2019 were included. The inclusion criteria were as follows: (1) diagnosis of acute ischemic stroke; (2) age ≥ 18 years; (3) large artery occlusion confirmed by computed tomographic angiography or digital subtraction angiography; (4) treatment with mechanical thrombectomy with or without intravenous alteplase according to the American Heart Association (AHA)–American Stroke Association (ASA) guidelines (1); and (5) hospitalization in Changhai Hospital after MT. The exclusion criteria were as follows: (1) diagnosis with community-acquired pneumonia; (2) other infectious diseases or treatment with broad-spectrum antibiotics or corticosteroid therapy for the 2 weeks before MT; (3) cancer; and (4) loss to follow-up. Written informed consent was obtained from all the participants. The study was approved by the Shanghai ethical committee.

### Diagnosis of PSP

Poststroke pneumonia was defined as a clinical diagnosis of pneumonia within 7 days after stroke onset during the hospital stay that did not fulfill the criteria for community-acquired pneumonia (3, 5), regardless of whether the patient was intubated. The clinical diagnosis of pneumonia was made based on the following findings: a new or progressive infiltrate, consolidation, or ground glass opacity revealed on chest computed tomography (CT) or radiography plus two or more of the following three criteria: (1) fever (>38°C) without another cause; (2) leukopenia (<4,000 leukocytes/mm^3^) or leukocytosis (>10,000 leukocytes/mm^3^); and (3) for patients older than 70 years old, at least two of the following: (a) a positive sputum culture; (b) new onset or worsening cough, or respiratory rate; (c) rales, crackles, or bronchial breath sounds; and (d) worsening gas exchange.

### Data collection and follow-up

Patient demographics, medical histories, laboratory findings, and clinical characteristics were extracted from the clinical records. These data included age, sex, body mass index (BMI = weight/height^2^), pre-stroke modified Rankin Scale (mRS) score, comorbidity, smoking, and drinking status, National Institutes of Health Stroke Scale (NIHSS) score, Glasgow Coma Scale (GCS) score, and systemic immune-inflammation index (SII, platelet × neutrophil/lymphocyte ratio) on admission, stroke onset to revascularization time (ORT), location of the lesion, the occlusion site, Alberta Stroke Program Early Computed Tomography Score (ASPECTS), treatment with intravenous alteplase, general anesthesia (GA) in the MT (including using GA at the beginning or converting from sedation during the MT operation), the presence of dysphagia, intubation after MT, and the degree of vessel recanalization after MT. The mRS at 90 days was used to evaluate the functional outcome, and an mRS score of ≤2 was considered a good outcome. Modified thrombolysis in cerebral infarction (mTICI) was used to measure vessel recanalization, of which mTICI ≥2b was defined as successful recanalization. All intracranial hemorrhage and symptomatic intracranial hemorrhage were diagnosed according to the Heidelberg criteria (8). Overall, two physicians blindly evaluated the imaging and procedural characteristics. If there was a disagreement, a third experienced physician made the final decision.

The patients were followed up by outpatient clinics or telephone at 90 days after stroke (within a window of ±14 days). The mRS at 90 days was used to evaluate the functional outcome, and mRS ≤2 was considered a good outcome. Trained physicians conducted interviews blindly. Mortality in the hospital and mortality at 90 days were included as safety outcomes.

### Statistical analysis

Statistical analysis was performed with SPSS Statistics version 25. Continuous variables with normal distribution were expressed as the means (SE), categorical variables were expressed as counts (%), and continuous variables not normally distributed were expressed as median (P_25_, P_45_) values. Differences between the groups were calculated using the *t*-test, the χ^2^-test, or the Mann–Whitney *U*-test as needed. Univariate analysis was used to identify the predictive parameters at a *p*-value of < 0.05. Variables with a *p-value of* < 0.05 in univariate analysis were included in the logistic regression analysis. In addition, *p* < 0.05 was considered statistically significant.

## Results

**Figure 1:**
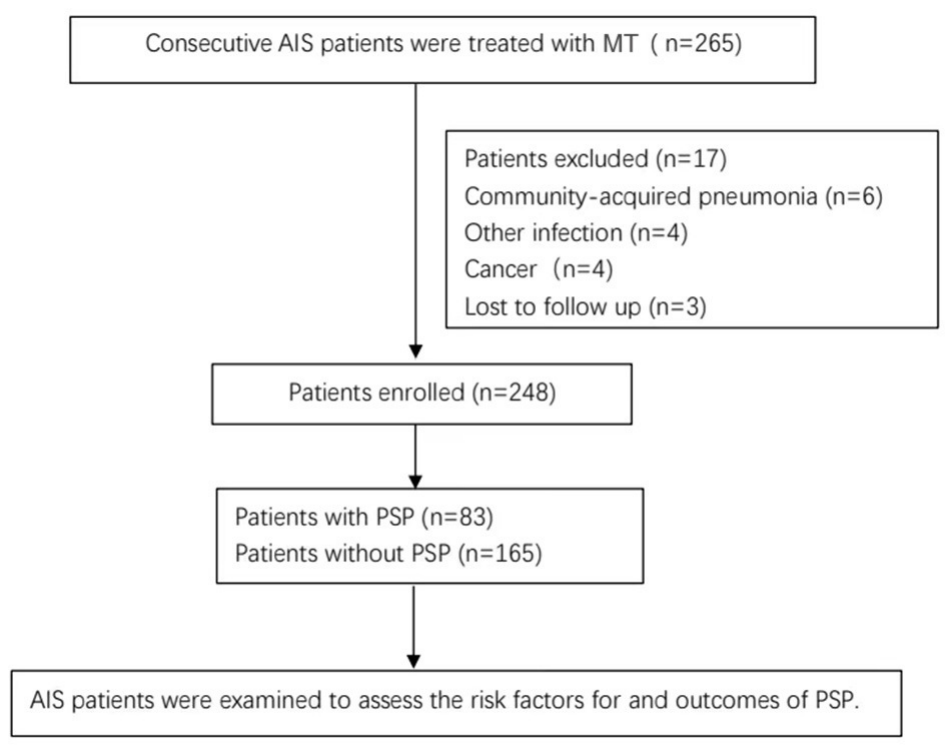
Enrollment flow diagram. AIS, acute ischemic stroke; MT, mechanical thrombectomy.

There were 265 patients with AIS treated with MT between January 2019 and December 2019 in our stroke center. In total, six patients were excluded because they were diagnosed with community-acquired pneumonia on admission, eight patients were excluded because they were diagnosed with other infectious diseases or cancer, and three patients were lost to follow-up. A total of 248 patients were included in the study (Figure 1), and 83 (33.47%) patients developed PSP. Of the 248 patients, the mean age was 67.19 ± 11.47 years, and 160 (64.5%) patients were men. The median time from stroke onset until the clinical diagnosis of PSP (marked by the prescription of antibiotics) was 2 days. The comparison of the patients with or without PSP is shown in Table 1. The univariate analysis suggested that BMI, hypertension history, serum glucose, SII, NIHSS score, GCS score, ASPECTS, white blood cell (WBC) count on admission, stroke ORT, NIHSS score at 24 h, median volume of lesions with CBF <30% on CT, dysphagia, intubation after MT, the posterior circulation AIS, and the use of sedatives after MT were significantly different between the PSP and non-PSP groups. The MT-related factors, stroke ORT, the median volume of lesions with CBF <30% on CT, and NIHSS score at 24 h after MT were significantly different between the two groups.

**Table 1:** Univariate analysis of predictive factors for developing poststroke pneumonia after mechanical thrombectomy.

**Variable**	**PSP**	**Non-PSP**	***p*-value**
Number	83	165	
Age, mean ± SD, y	68.63 ± 11.03	66.47 ± 11.64	0.163
Male, no. (%)	57 (68.67)	103 (62.42)	0.332
BMI, median (P_25_, P_75_), kg/m^2^	24.22 (22.85, 27.14)	23.67 (21.48, 25.95)	0.035
Hypertension, no. (%)	64 (77.11)	107 (64.85)	0.049
Diabetes mellitus, no. (%)	28 (33.73)	47 (28.48)	0.396
Atrial fibrillation, no. (%)	36 (43.37)	62 (37.58)	0.378
Prestroke mRS score ≤2, no. (%)	82 (98.80)	164 (99.39)	0.619
BNP, mean ± SD, pg/mL	226.79 ± 50.26	167.54 ± 22.82	0.227
Current smoker, no. (%)	34 (40.96)	68 (41.21)	0.970
Drinker, no. (%)	20 (24.10)	36 (21.82)	0.686
Hyperlipemia, no. (%)	15 (18.07)	28 (16.97)	0.859
Coronary heart disease, no. (%)	7 (8.43)	28 (16.97)	0.180
NIHSS score on admission, median (P_25_, P_75_)	19 (16, 22)	16 (11, 20)	<0.001
GCS score on admission, median (P_25_, P_75_)	11 (9, 12)	16 (11, 20)	0.001
Serum glucose on admission, mean ± SD, mmol/L	8.87 ± 3.72	7.86 ± 3.07	0.031
Serum creatinine on admission, mean ± SD, mmol/L	73.86 ± 21.38	76.20 ± 20.90	0.429
SII on admission, mean ± SD	1,619.49 ± 1,226.74	1,006.68 ± 887.93	<0.001
WBC on admission, mean ± SD	9.81 ± 3.36	8.70 ± 3.09	0.011
ASPECTS before treatment, median (P_25_, P_75_)	7.50 (4.00, 9.00)	9.00 (7.00, 10.00)	0.002
Side of target occlusion			0.865
Left, no	43	81	
Right, no	33	67	
Bilateral, no	7	17	
Location of intracranial artery occlusion, no			0.069
Intracranial ICA, no	23	41	
MCA M1 segment, no	19	63	
MCAM2 segment, no	11	17	
Basilar	17	17	
Tandem lesions, no			0.699
BA with VA occlusions	1	6	
MCA M1 with the other variable occlusions	6	13	
Other variable tandem occlusion	3	6	
Intravenous thrombolysis, no. (%)	5 (6.02)	11 (6.67)	0.846
Posterior circulation AIS, no. (%)	21 (25.3)	22 (13.9)	0.027
ORT, median (P25, P75)	456.00 (313.00, 687.00)	373 (260.00, 550.00)	0.008
General anesthesia, no. (%)	54 (65.06)	88 (53.33)	0.078
Intubation after MT, no. (%)	42 (50.60)	16 (9.70)	<0.001
Sedatives after MT, no. (%)	20 (24.10)	12 (7.27)	<0.001
mTICI ≥ 2b, no. (%)	79 (95.18)	160 (96.97)	0.477
Dysphagia, no. (%)	72 (86.75)	49 (29.70)	<0.001
Intracranial hemorrhage, no. (%)	33 (39.76)	46 (27.88)	0.058
Symptomatic intracranial hemorrhage, no. (%)	11 (13.25)	11 (6.67)	0.085
Volume of lesions with CBF <30% on CT, median (P_25_, P_75_), cm^3^	25 (0.65)	7 (0.22)	0.001
NIHSS score at 24 hours, median (P_25_, P_75_)	19 (13.26)	5 (2.13)	<0.001
Cardiac embolism, no. (%)	32 (38.55)	62 (37.58)	0.970
Use of PPIs, no. (%)	68 (81.92)	134 (81.21)	0.891
Use of H2RA, no. (%)	18 (21.68)	34 (20.61)	0.844

AIS, acute ischemic stroke; MT, mechanical thrombectomy; PSP, poststroke pneumonia; BMI, body mass index; mRS: modified Rankin scale; BNP, brain natriuretic peptide; NIHSS, National Institutes of Health Stroke Scale; GCS, Glasgow Coma Scale; SII, systemic immune-inflammation index; ASPECTS, Alberta Stroke Program CT Score; ICA, internal carotid artery; MCA, middle cerebral artery; BA, basilar artery; VA, vertebral artery; ORT, stroke onset to revascularization time; GA, general anesthesia; mTICI, modified thrombolysis in cerebral infarction; CBF, cerebral blood flow; H2RA, histamine 2 receptor antagonist.

Variables with *p* < 0.05 in univariate analysis were included in the logistic regression model. The independent predictors of PSP were BMI (OR 1.200, 95% CI 1.038–1.387; *p* = 0.014), SII (OR 1.001, 95% CI 1.000–1.002; *p* = 0.003), dysphagia (OR 9.498, 95% CI 3.217–28.041; *p* < 0.001), and intubation after MT (OR 4.262, 95% CI 1.166–15.581; *p* = 0.028). The details are shown in Table 2.

**Table 2:** Logistic regression analysis of possible predictive factors of developing poststroke pneumonia after mechanical thrombectomy.

**Parameter**	**OR**	**95% CI of Exp (B)**		***p***
BMI	1.200	1.038	1.387	0.014
Hypertension	0.897	0.321	2.508	0.835
GCS score on admission	1.165	0.921	1.474	0.204
NIHSS score on admission	1.026	0.963	1.092	0.428
Serum glucose on admission	0.947	0.827	1.085	0.434
SII on admission	1.001	1.000	1.002	0.003
ASPECTS on admission	0.965	0.757	1.229	0.771
ORT	1.000	0.998	1.001	0.574
Intubation	4.262	1.166	15.581	0.028
NIHSS at 24 h	1.026	0.963	1.092	0.428
Median volume of lesions with CBF <30% on CT	1.002	0.988	1.016	0.763
WBC on admission	0.884	0.763	1.025	0.102
Dysphagia	9.498	3.217	28.041	0.000
Sedatives	1.708	0.506	5.759	0.388
posterior circulation AIS	0.384	0.099	1.492	0.167

Variables with P <0.05 in logisticregression were included in the logisticregression analysis.

BMI, body mass index; SII, systemic immune-inflammation index; AIS, acute ischemic stroke.

**Figure 2:**
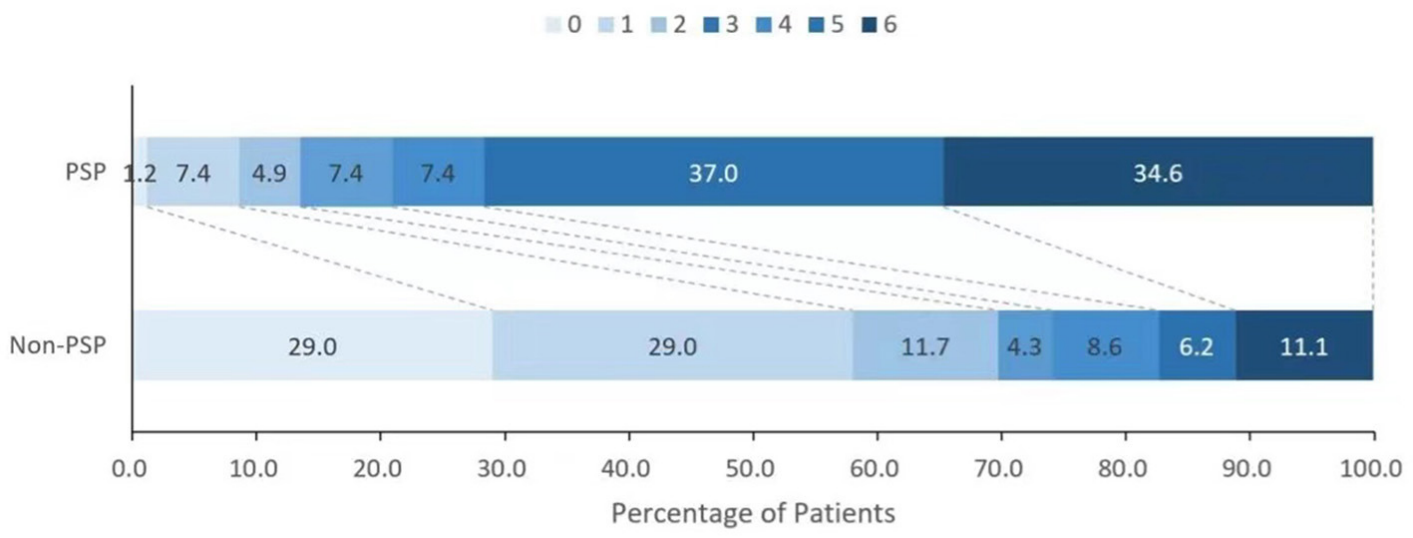
Distribution of the modified Rankin scale scores between patients with and without poststroke pneumonia.

**Figure 3:**
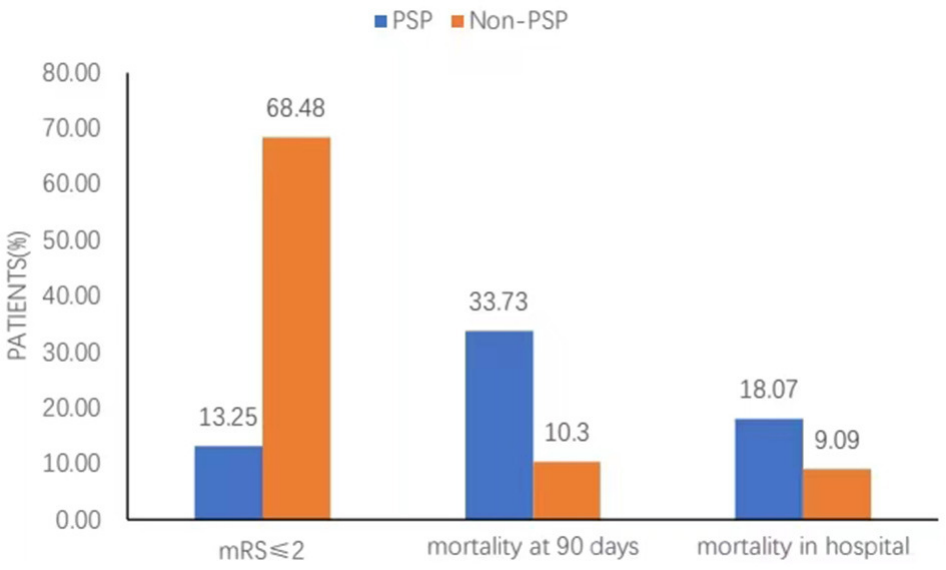
Distribution of clinical outcomes in-hospital and at 90 days.

The overall 90-day mortality of the cohort was 18.6%, and this rate was associated with the presence of PSP. Compared with the patients with non-PSP, the patients with PSP had a higher in-hospital mortality (18.07 vs. 9.09%; *p* = 0.061) and 90-day mortality (33.73 vs. 10.30%; *p* < 0.001) and had a lower functional independence rate (mRS score ≤2) (13.25 vs. 68.48%; *p* < 0.001) (Figures 2, 3). PSP was negatively associated with functional independence (mRS score ≤2) (OR 0.104, 95% CI 0.041–0.260; *p* < 0.001) and positively associated with 90-day mortality (OR 3.010, 95% CI 1.068–8.489; *p* = 0.037) (Table 3).

**Table 3:** Impacts of poststroke pneumonia on clinical outcomes in the multivariate model.

**Outcomes**	**Unadjusted**			**Adjusted**		
	**OR**	**95% CI**	***P***	**OR**	**95% CI**	***P***
In-hospitalmortality	2.689	1.183–6.113	0.018	1.464	0.478–4.483	0.504
90-day mortality	4.475	2.267–8.833	<0.001	3.010	1.068–8.489	0.037
Functional independence (mRS score ≤2)	0.067	0.032–0.137	<0.001	0.104	0.041–0.260	<0.001

The logistic regression model included the factors NIHSS score, GCS score, ASPECTS on admission, the median volume of lesions with CBF <30%, ORT, and mTICI.

NIHSS, National Institutes of Health Stroke Scale; GCS, Glasgow Coma Scale; ASPECTS, Alberta Stroke Program Early CT Score; ORT, stroke onset to revascularization time; mTICI, modified thrombolysis in cerebral infarction.

## Discussion

The incidence of PSP after AIS varies from 5.4 to 44% depending on the clinical setting and the definition based on the literature (9, 10). In our study, 33.47% of the patients with AIS treated with MT developed PSP, which was significantly higher than the ~ 12% incidence of pneumonia in patients who have had AIS of any type (9).

Moreover, our study demonstrated that PSP was negatively associated with functional independence (mRS ≤2) and was positively associated with mortality rates in patients with AIS who received MT. Similar findings were also reported in many other studies (11). Infection is already considered a determinant of outcomes after stroke (12). Among all infections, pneumonia had the greatest impact on the outcome of patients with stroke. The proportion of deaths attributed to pneumonia occurring within the 1st week after stroke onset accounted for one-third of all deaths in patients with acute ischemic stroke. A nationwide 4-year study confirmed that the patients with hospital-acquired pneumonia (HAP) after the stroke had an elevated risk of death (OR, 1.2; 95% CI, 1.1–1.3) (13). Our research subjects had PSP, including not only HAP but also ventilator-associated pneumonia (VAP) after stroke. PSP significantly elevated 90-day mortality (OR 3.010; 95% CI, 1.068–8.489) in our study. Therefore, identifying risk factors and the early identification of PSP are important for patients with AIS treated with MT.

This study demonstrated that patients with higher BMI, higher SII on admission, dysphagia, and intubation after MT were more likely to develop PSP. As risk factors for PSP, dysphagia and intubation after MT have been frequently reported by various studies (8, 11). This indicates that the predictive risk factors remain mostly identical in patients with AIS treated with MT. However, they suffer more severe stroke symptoms and have an overall higher risk of developing PSP. Intubation due to the severity of the stroke, not due to GA, is an independent risk factor for PSP, which indicates that the severity of the stroke itself is the key point for PSP. The NIHSS of patients with PSP was higher than that of patients with non-PSP (*p* < 0.001) but was not an independent factor for PSP according to logistic regression. This may be because other stroke factors affecting respiration directly, such as dysphagia and intubation, are more closely associated with PSP in patients with AIS with MT (5). Another reason may be that the sample size in our study was also small. The effect of anesthesia on pneumonia has always been a controversial issue. Our study did not find that GA was an independent risk factor for PSP. Many previous studies and meta-analyses have confirmed that there is no difference in complications and outcomes after stroke between patients who underwent GA and those who underwent conscious sedation (7, 14, 15).

Interestingly, we found that BMI was also an independent predictor of PSP. One study reported that obesity was a predictor of an increased risk of in-hospital complications in patients with cerebral hemorrhage (16). Another study reported that for patients treated with MT, a high BMI was independently associated with lower rates of functional independence among recanalized patients (17). A meta-analysis also demonstrated an association between obesity and increased postoperative complications (18). However, the relationship between obesity and stroke outcomes is still unclear. A *post-hoc* analysis of the MR CLEAN trial found that obesity was associated with better functional outcomes after a stroke in patients treated with MT (19). The NIH FAST-MAG study reported that obesity was associated with increased survival but had a U-shaped or J-shaped relation to disability and stroke-related quality of life (20). Although BMI was an independent factor affecting PSP, the BMI values did not differ between the patients with good and poor outcomes in our study. Higher quality evidence is needed to clarify the relationship between obesity and outcome in patients with stroke.

The SII, which combines platelets, lymphocytes, and neutrophils to reflect thrombosis and inflammation, was also an independent predictor of PSP based on our study. The inflammatory mechanism after stroke plays a critical role in the development of AIS ischemic stroke (21). Stroke induces the activation of the inflammatory cascade in both the CNS and PNS, which is called stroke-induced immunosuppression (SIIS). It occurs within hours of stroke onset and increases the host's susceptibility to poststroke infection. The inflammatory response of AIS is complex. The damage caused by neutrophils causes systemic inflammation and damages the blood–brain barrier. Platelets become excessively active and begin to accumulate. Inflammatory cytokines trigger lymphocyte apoptosis (22). The infiltration of leukocytes and the release of various inflammatory mediators may also result in adverse outcomes. A high SII was an independent risk factor for poor outcomes at 3 months in patients with AIS (23, 24). Ahmet Adiguzel et al. reported that the SII reached statistical significance in terms of discriminability for pneumonia development (25) based on daily measurement of the SII. Our study only recorded the SII on admission but still indicated an association with PSP. Therefore, the SII, which is a relatively integrated index that can be quickly calculated from blood, maybe a potential prognostic factor for PSP in clinical practice. Large-scale population data are needed to verify the reliability of SII in predicting PSP.

The study had some limitations. First, as a retrospective single-center study, the sample size was small, which may lead to selection bias. Second, the degree of dysphagia and feeding actions were not analyzed. Third, only SII on admission was calculated. The correlation between the dynamic monitoring of SII and PSP was unclear. The associations between PSP and other inflammatory markers, such as C-reactive protein, were not discussed in this study. Finally, our study included all patients with pneumonia except the patients with community-acquired pneumonia and did not distinguish ventilator-associated pneumonia.

## Conclusion

This study found that nearly one in three patients with stroke with AIS treated with MT developed PSP during acute care. Patients with AIS who developed PSP had worse outcomes than those without PSP. Dysphagia, intubation after MT, higher BMI, and SII on admission were all associated with PSP in these patients. The findings of the current study may help to prevent the development of PSP by identifying patients who are at risk.

## Data availability statement

The raw data supporting the conclusions of this article will be made available by the authors, without undue reservation.

## Ethics statement

The studies involving human participants were reviewed and approved by the Ethics Committee of the First Affiliated Hospital of Naval Medical University (M2019-010-2019-12-10). The patients/participants provided their written informed consent to participate in this study. Written informed consent was obtained from the individual(s) for the publication of any potentially identifiable images or data included in this article.

## Author contributions

PZ and LC contributed to draft the manuscript. HY and YJ contributed to collect the data. XZ and MZ contributed to follow the patients. TW, BD, and JL contributed to polish the language. PY and YZ contributed to revise the manuscript. All authors contributed to the article and approved the submitted version.
